# Early Sodium Dynamics in Severely Injured Patients with Intestinal Perforations: A Multicenter Analysis

**DOI:** 10.3390/jcm15186984

**Published:** 2026-09-09

**Authors:** Nicolas Bless, Simon Tiziani, Christian Maschmann, Henrik Teuber, Beat Schnüriger, Marlene Mauch, Andreas Marc Müller, Swiss Trauma Registry

**Affiliations:** 1 Department of Orthopedics and Traumatology, University Hospital Basel, 4031 Basel, Switzerland; simon.tiziani@usb.ch (S.T.); marlene.mauch@usb.ch (M.M.); a.mueller@usb.ch (A.M.M.); 2 Notfallzentrum NFZ, Health Ostschweiz HOCH, University of St. Gallen, 9000 St. Gallen, Switzerland; christian.maschmann@h-och.ch; 3 EMS Regio144, 8630 Rüti, Switzerland; 4 Department of Traumatology, University Hospital Zurich, 8091 Zurich, Switzerland; henrik.teuber@usz.ch; 5 Department of Visceral Surgery and Medicine, Inselspital, Bern University Hospital, 3010 Bern, Switzerland; beat.schnueriger@insel.ch; 6 Swiss Trauma Board, 8091 Zürich, Switzerland

**Keywords:** hyponatremia, polytrauma, abdominal injuries, diagnostics, intestinal perforation

## Abstract

**Background:** Diagnosis of intestinal perforation in severely injured patients remains challenging. Hyponatremia has been associated with intestinal inflammation in other clinical settings. The aim of this study was to determine whether severely injured patients with an intestinal perforation present lower serum sodium levels compared to severely injured patients without perforation. **Methods:** A retrospective cohort study was conducted using data from the Swiss Trauma Registry (STR), including 72 severely injured patients with and 61 without intestinal perforation. Serum sodium levels at admission and immediately prior to surgery were retrieved from medical records. Differences in sodium levels were evaluated using linear mixed-effects models to account for repeated measures and group effects (*p* < 0.05). **Results:** There was no significant difference in sodium levels between patients with an intestinal perforation compared to controls (mean difference: +0.74 mmol/L, *p* = 0.204). The time × group interaction indicated a slightly greater decline in sodium from first to last assessment in the perforation group relative to controls (−1.14 mmol/L, *p* = 0.062); however, these effects were not statistically significant. **Conclusions:** No significant difference in sodium levels between the patients with and without intestinal perforation undergoing laparotomy in the early phase of hospitalization was found that would have potentially allowed for diagnosis of a hollow viscus organ injury.

## 1. Clinical Case

**Figure 1:**
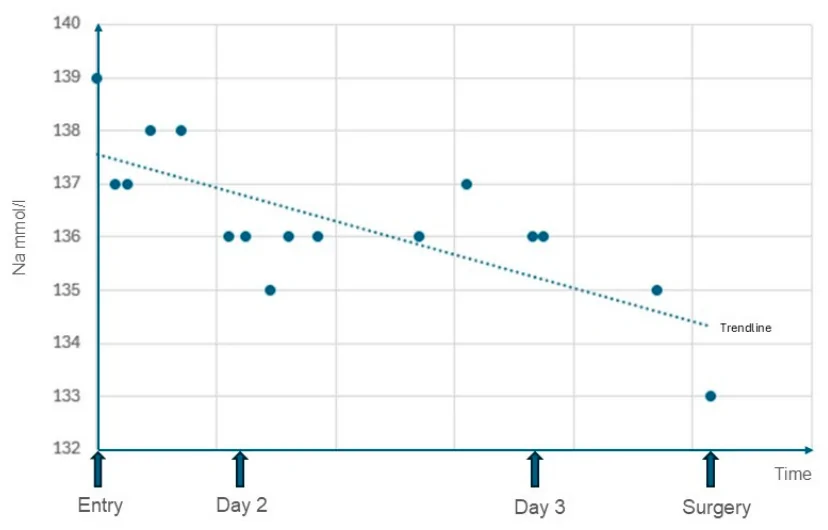
Dynamics of sodium levels in 21-year-old female patient with perforations of jejunum.

A 21-year-old female car driver suffered a high-energy trauma from a head-on collision with another car. She was extricated from the wreck by fire fighters and transferred to a level 1 trauma center in Switzerland as an acute trauma transfer. Clinical assessment showed a minor trauma to the head, a pneumothorax, a blunt abdominal trauma and an open femur fracture. Initial abdominal CT demonstrated a small amount of free fluid, mesenteric edema, and small-bowel wall thickening, but no evidence of active bleeding or hard signs of a bowel perforation. Following surgery for the open fracture, the patient was transferred to the ICU. Two days after the accident the patient developed an acute abdomen, underwent exploratory laparotomy and perforations of the jejunum were found. Serum sodium level at admission was 140 mmol/L and declined steadily, reaching 133 mmol/L before laparotomy (Figure 1). Kidney function estimated by creatinine clearance calculated by Cockcroft–Gault equation remained stable between 133 mL/min and 128 mL/min.

## 2. Introduction

Missed intra-abdominal injuries are among the most frequent causes of potentially preventable trauma deaths [1]. In particular, hollow viscus injuries have a more insidious presentation than solid organ injuries and are associated with a delay to diagnosis [2]. Early identification of these injuries remains challenging. A timely diagnosis is crucial to reduce morbidity and mortality in severely injured patients. Blunt abdominal trauma outnumbers penetrating trauma by a large margin in Switzerland [3]. Perforations due to blunt trauma are harder to catch, as both clinical exam and computed tomography results can be unreliable. In a large series by Agri et al., up to 18% of patients with bowel and mesenteric injuries had delays to surgery of >24 h despite having undergone a computed tomography scan [4]. Difficulty in diagnosing intestinal perforations in blunt trauma is exacerbated by the trend of treating abdominal solid organ injuries non-operatively. In the past, one was able to discover perforations during exploratory laparotomy for other causes more routinely. Such opportunity is increasingly missing due to the successful expectant management of most solid organ injuries.

There is no one gold-standard test to rule in or rule out intestinal perforation. The treating trauma team has to rely on clinical findings, hard and soft signs on computed tomography scans, and, if warranted, surgical exploration to confirm or rule out the presence of a traumatic intestinal perforation. Peritonitis may be present and clinical signs such as a rigid abdomen, guarding or rebound tenderness can help the treating team raise suspicion for an acute intra-abdominal pathology. However, delayed onset of peritoneal signs, distracting injuries and clinically hard-to-assess trauma patients contribute to the uncertainty when trying to rule out intestinal perforation. Although peritoneal signs are highly specific for a trauma patient requiring exploratory surgery, the sensitivity for traumatic intestinal perforation is poor [5].

Sensitivity for intestinal perforation in penetrating trauma can be significantly improved if the patient is stable enough to undergo an abdominal computed tomography scan [6]. Hard signs with high specificity for a perforation include: a pneumoperitoneum or mesenteric air, extraluminal oral contrast and clear bowel discontinuity seen on imaging [7]. In keeping with clinical signs, these radiographical signs alone offer poor sensitivity in isolation. Sensitivity for blunt trauma patients can be as low as 80% [8]. Thus, a rule-out of an intestinal perforation in the trauma bay is often unrealistic and patients need to be followed with serial exams. Additional objective tests indicating the red flags would be most welcome.

Hyponatremia has previously been described as a predictor of ischemic–necrotic infections, i.e., gangrenous cholecystitis and as a specific marker of infectious colon perforation [9,10,11,12]. In colorectal surgery, decreased serum sodium levels have been proposed as a potentially negative predictive marker for anastomotic leakage [13]. Although the underlying mechanisms are not fully understood, hyponatremia is recognized as a complication of several inflammatory diseases [14]. In most cases, it is attributed to non-osmotic vasopressin (antidiuretic hormone) secretion [14], triggered by inflammatory stimuli, hypovolemia, pain, nausea, and various medications [14,15]. Vasopressin primarily regulates water homeostasis and serum osmolarity but also acts as an endogenous stress hormone with vasoconstrictive effects through arginine vasopressin 1A receptors, particularly during shock [16].

Based on this evidence, similar sodium dysregulation could serve as a physiological signal of intestinal perforation in severely injured patients. However, to date no study has specifically investigated the association between serum sodium levels and intestinal perforations in severely injured patients. Identifying such a biomarker could support earlier detection, enable more rapid clinical decision-making, and ultimately contribute to improved outcomes and reduced trauma-related mortality.

The aim of our study was therefore to assess the relationship between serum sodium levels and intestinal perforations in severely injured patients. We hypothesized that patients with intestinal perforations would present with lower sodium levels compared to a control group.

## 3. Materials and Methods

### 3.1. Study Design

This retrospective cohort study utilized data from the Swiss Trauma Registry (STR) for the period between June 2015 and September 2024. The STR is a national, multicenter database. It provides standardized and pseudonymized documentation of prospectively collected data on patients with severe injuries treated at twelve participating trauma centers in Switzerland. For the present analysis, four centers contributed data.

Severe injury in the STR is defined as an injury severity score (ISS) ≥ 16 and/or an abbreviated injury scale (AIS) head score ≥ 3. The registry includes information on prehospital care, emergency department management, initial surgical interventions, intensive care unit treatment, and hospital discharge.

Patients were eligible for inclusion if their STR data contained an AIS code indicating a perforation of the small intestines or colon (codes 5408xx, 5410xx, 5414xx) [17]. Serum sodium levels at admission and immediately prior surgery are not part of the routinely collected STR dataset; therefore, these values were additionally retrieved from electronic medical records at each participating center.

**Figure 2:**
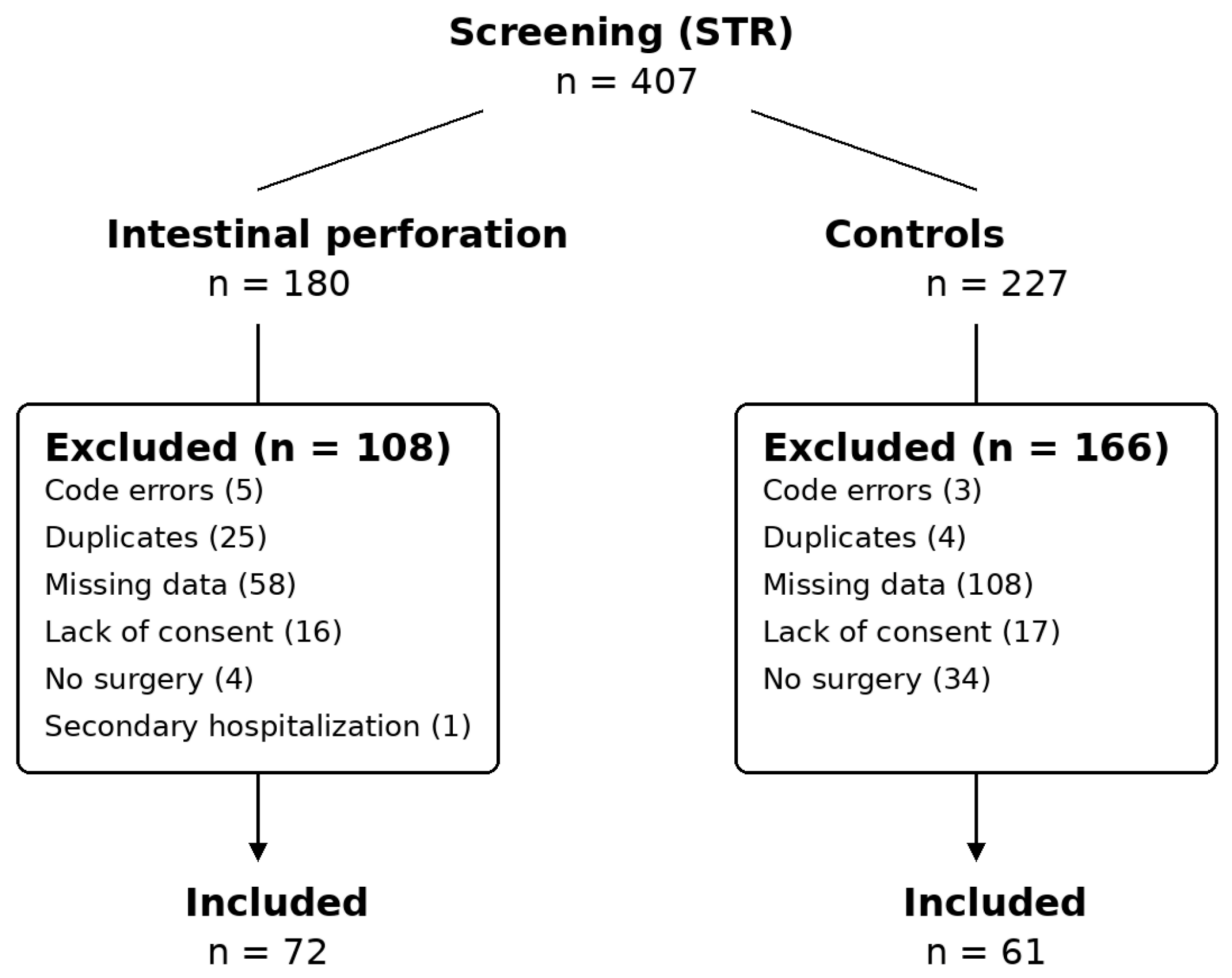
Flow Chart of patients’ inclusion.

Patients were compared to a control cohort consisting of randomly selected STR patients without intestinal perforations undergoing any other type of surgery. Patients without any documented serum sodium measurement within 24 h prior to surgery were excluded from the analysis (Figure 2). The study was conducted under STR-Project-ID 17 and was approved by the Swiss Trauma Board in accordance with the STR publication guidelines, as well as by the regional ethics committee (BASEC-2024-00892).

### 3.2. Data Analysis

The primary endpoints were the initial serum sodium level measured on admission and the last serum sodium level obtained immediately before surgery. To address the question of whether intestinal perforation has an impact on the development of sodium, linear mixed models were applied. These models incorporated all sodium assessments as the dependent variable and interaction between initial and last measurements (time) and intestinal perforation versus the control group (group) as a fixed effect. The patient identification number was incorporated as a random effect. This analytical approach encompassed two measurements per patient.

Continuous variables are presented as median with interquartile range (IQR), and group differences were compared using the Wilcoxon–Mann–Whitney test. Categorical variables are reported as counts and percentages, and group differences were analyzed using Fisher’s exact test. All statistical analyses were performed using StataNow/SE 19.5 (Stata Corp., College Station, TX, USA).

## 4. Results

### 4.1. Patients’ Characteristics

A total of 133 patients fulfilled the eligibility criteria and were included in the analysis, comprising 72 patients with an intestinal perforation and 61 control patients without perforation. Baseline demographic and injury characteristics were generally comparable between groups (Table 1). Median age was 50 years (IQR 28–59) in the perforation group and 54 years (IQR 36–63) in the control group (*p* = 0.111). Male patients predominated in both cohorts, accounting for 82% of patients with intestinal perforation and 72% of controls (*p* = 0.212). Injury severity was substantial in both groups, with median ISS values of 24 (IQR 18–34) and 27 (IQR 24–34), respectively, without statistically significant differences (*p* = 0.071).

Physiological injury severity at admission, assessed using the weighted Revised Trauma Score (RTS), was comparable between groups. Among patients with complete physiological data available, the median RTS was 7.33 (IQR 6.66–7.84) in the perforation group and 7.23 (IQR 6.90–7.84) in the control group, with no significant difference observed (*p* = 0.648). Despite comparable injury severity at presentation, patients with intestinal perforation experienced significantly longer hospital stays, with a median length of stay of 18 days (IQR 13–24) compared with 11 days (IQR 7–17) among controls (*p* < 0.001), suggesting a greater overall burden associated with these injuries.

### 4.2. Sodium Levels

Serum sodium concentrations at admission and immediately before surgery were similar between the two groups (Table 2). At hospital admission, median sodium levels were 140 mmol/L (IQR 137–142) in patients with intestinal perforation and 139 mmol/L (IQR 136–140) in controls (*p* = 0.251). Prior to surgery, sodium concentrations remained comparable, with median values of 139 mmol/L (IQR 136–140) and 139 mmol/L (IQR 137–141), respectively (*p* = 0.371). Overall, sodium concentrations remained within the physiological range in both groups throughout the observation period.

With respect to timing of operative intervention, patients with intestinal perforation underwent surgery significantly earlier during their hospitalization than controls. The median interval between admission and surgery was 1 day (IQR 1–1) in the perforation group compared with 1 day (IQR 1–3) in the control group (*p* < 0.001).

### 4.3. Longitudinal Sodium Analysis

To account for repeated sodium measurements over time, a linear mixed-effects model was applied (Table 3). No statistically significant main effect of intestinal perforation on serum sodium concentration was observed. Across both measurement time points, patients with intestinal perforation demonstrated sodium values that were on average 0.74 mmol/L higher than controls (95% CI −0.40 to 1.89; *p* = 0.204).

The interaction analysis revealed a trend towards a greater decline in sodium concentrations over time among patients with intestinal perforation compared with controls. Specifically, sodium levels decreased by an additional 1.14 mmol/L in the perforation group relative to controls (95% CI −2.33 to 0.06; *p* = 0.062) (Table 3).

**Figure 3:**
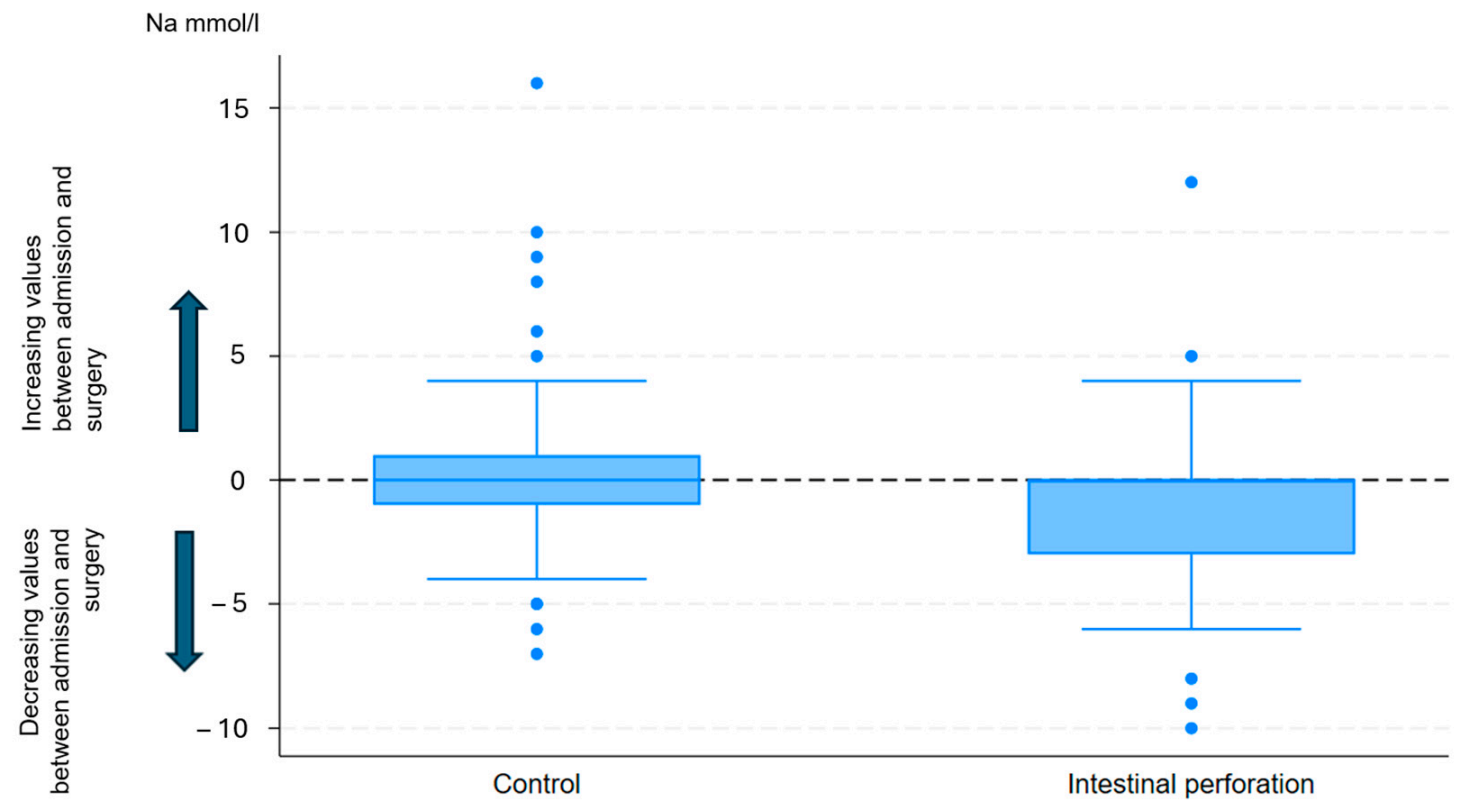
Change in serum sodium levels between admission and immediately prior to surgery in patients with intestinal perforation (**right**) and controls (**left**); positive values indicate an increase in sodium levels, negative values indicate a decrease in sodium levels.

Individual patient trajectories demonstrated substantial inter-individual variability in sodium levels between admission and surgery (Figure 3). While both increases and decreases in sodium levels were observed in each group, patients with intestinal perforation exhibited a tendency towards more negative sodium changes. Nevertheless, considerable overlap between groups was present, preventing reliable discrimination based on sodium dynamics alone.

## 5. Discussion

In this multicenter cohort of severely injured patients undergoing surgery at an early stage of hospitalization, no significant association was observed between serum sodium levels and the presence of traumatic intestinal perforation. Patients with intestinal perforation demonstrated sodium concentrations comparable to those of severely injured controls both at admission and immediately prior to surgery. Although a trend towards a greater decline in sodium levels was observed in patients with perforation, the magnitude of this effect remained small and did not reach statistical significance.

The present findings provide important evidence regarding the potential role of serum sodium as an adjunct diagnostic marker for bowel injury after trauma. Previous investigations in non-traumatic gastrointestinal disease have suggested that hyponatremia may be associated with bowel ischemia, complicated appendicitis, perforated diverticulitis, incarcerated hernias, and anastomotic leakage [10,11,12,13,14,15,16,18]. In these conditions, hyponatremia is thought to result from a combination of inflammatory cytokine release, non-osmotic vasopressin secretion, hypovolemia, gastrointestinal fluid losses, and alterations in renal water handling. Interleukin-6 and other inflammatory mediators have been shown to stimulate vasopressin release independently of serum osmolality, thereby promoting water retention and dilutional hyponatremia [14,15,16].

The biological rationale for investigating sodium levels in traumatic intestinal perforation is therefore plausible. Perforation of the bowel initiates local and systemic inflammatory responses, peritoneal contamination, fluid shifts into the third space, and activation of neuroendocrine stress pathways [19]. These processes lead to progressive sodium dilution similar to that observed in other inflammatory gastrointestinal disorders [10,18]. Indeed, our illustrative clinical case demonstrated a gradual decline in serum sodium levels beginning approximately two days after injury, while renal function remained stable.

Since electrolyte changes develop over time, we consider time to be the key factor explaining the discrepancy between our findings and those reported in non-traumatic gastrointestinal diseases. Most patients in the present cohort underwent operative intervention very early after admission, with a median interval of only one day before surgery. Consequently, the duration of ongoing inflammation before definitive treatment was relatively short. In contrast, many patients included in studies of diverticulitis, appendicitis, bowel ischemia, or anastomotic leakage experience prolonged inflammatory processes over several days before diagnosis and treatment. The shorter inflammatory exposure in trauma patients may limit the development of measurable sodium disturbances and thereby reduce the diagnostic utility of this parameter in the acute setting.

In contrast, severely injured patients with blunt abdominal trauma undergo immediate clinical and radiological evaluation upon hospital admission to identify intestinal injuries, including perforations. The introduction of multi-detector computed tomography (MDCT) has represented one of the most significant advances in trauma imaging in recent decades [20]. However, the presence of free intraperitoneal air found on abdominal CT scans in blunt trauma has been shown to be an unreliable radiological finding for predicting bowel perforation [21,22]. In the setting of free intraperitoneal air, findings that were highly predictive of intra-abdominal injury were free fluid and radiographic signs of bowel trauma, as well as the clinical and/or radiographic seatbelt sign [22]. When no free intraperitoneal air is detected and only nonspecific signs of bowel and mesenteric findings are present on CT, correlation with clinical findings remains essential, and a repeat CT scan after 6–8 h may help to determine the significance of these equivocal findings [23]. Given that severely traumatized patients frequently require prolonged intubation and are not readily assessable clinically, an additional quantifiable parameter to aid in the diagnosis of hollow viscus perforations, especially in obtunded patients, may be of clinical value. While serum sodium levels tended to decrease prior to surgery in patients with perforation, this difference did not reach statistical significance compared with controls in our cohort, possibly due to the early timing of laparotomy.

Trauma is a significant confounder when evaluating serum sodium as a marker for intestinal perforation. In severely injured patients, numerous factors unrelated to bowel injury can contribute to hyponatremia and influence sodium homeostasis. Hypovolemia due to hemorrhage triggers non-osmotic vasopressin release and renal water retention, resulting in dilutional hyponatremia, an effect that may be further amplified by large-volume crystalloid resuscitation, blood product administration, and third-space fluid losses [24]. In addition, traumatic brain injury can lead to hyponatremia through the syndromes of inappropriate antidiuretic hormone secretion (SIADH) or cerebral salt wasting syndrome [25].

Other trauma-related factors, including hemorrhagic shock, vasopressor therapy, pain-induced stress responses, and intensive care management, may also affect sodium regulation independently of intestinal perforation. Collectively, these competing mechanisms can substantially increase variability in serum sodium concentrations and potentially mask subtle changes attributable specifically to bowel perforation. Consequently, serum sodium alone is unlikely to provide sufficient diagnostic accuracy as a stand-alone marker in the acute trauma setting. Future research should therefore focus on multimodal diagnostic strategies that integrate laboratory parameters, clinical findings, and imaging features rather than evaluating sodium levels in isolation [6,7,23].

Importantly, the absence of a significant association also represents a clinically relevant finding. Serum sodium is universally available and inexpensive, making it an attractive candidate biomarker. However, our results suggest that routine sodium measurements should not be used to support or exclude the diagnosis of traumatic intestinal perforation during the early phase after injury. Clinical judgment, serial examination, and radiological assessment remain the cornerstones of diagnosis in these patients.

This study has several limitations. The retrospective design limits the strength of the conclusions and reduces statistical power. It is also important to note the limited sample size and restricted recruitment of trauma centers. Although we were able to include the two largest trauma centers in Switzerland, only 4 of the 12 eligible centers participated in the study.

Furthermore, the registry-based design limits the availability of several clinically relevant variables. In particular, information regarding the presence and extent of peritonitis was not systematically collected, as patient inclusion was based on AIS injury codes reflecting structural bowel damage rather than clinical manifestations. Consequently, we were unable to stratify patients according to the presence or severity of peritoneal contamination or to evaluate whether sodium dynamics differed between patients with and without peritonitis. Similarly, detailed data regarding perioperative sodium replacement and fluid management were not available, precluding assessment of their potential influence on serum sodium concentrations. Complete data for calculation of the weighted RTS were only available in a subset of patients, which may have limited the assessment of physiological injury severity and comparability between groups.

Further, most patients in both groups underwent early surgery shortly after admission, potentially before the development of a pronounced inflammatory response capable of inducing hyponatremia. As a result, the relationship between trauma, inflammation, and sodium dysregulation could not be fully captured. Finally, we were unable to account for pre-trauma renal function because these data were unavailable.

Future prospective studies with serial sodium measurements, standardized assessment of peritonitis severity, and detailed documentation of fluid and electrolyte management, particularly in patients undergoing laparotomy for intestinal perforation at later stages, are needed to further elucidate this potential association and its clinical applicability.

## 6. Conclusions

No association between decreased serum sodium levels and the presence of intestinal perforation was observed in this cohort of severely injured patients undergoing early laparotomy; accordingly, hyponatremia cannot be used as an early-phase indicator following trauma. Further studies including patients treated at later stages are warranted to better elucidate its potential role.

## Figures and Tables

**Table 1:** Patient characteristics.

	Total (*n* = 133)	Control (*n* = 61)	Intestinal Perforation (*n* = 72)	*p*
Age (years)	51 (31 to 61)	54 (36 to 63)	50 (28 to 59)	0.111 ^1^
Gender (Male/Female)	103/30 (77/23%)	44/17 (72/28%)	59/13 (82/18%)	0.212 ^2^
Injury Severity Score (ISS)	27 (21 to 34)	27 (24 to 34)	24 (18 to 34)	0.071 ^1^
Revised Trauma Score (RTS)	7.33 (6.84–7.84)	7.23 (6.90–7.84)	7.33 (6.66–7.84)	0.648
Length of Stay (LOS) (days)	15 (9.0 to 23)	11 (7.0 to 17)	18 (13 to 24)	<0.0011 ^1^

Values are presented as median (IQR) for continuous variables and counts (percentages) for categorical variables. Tests used: ^1^ Mann–Whitney U test. ^2^ Fisher’s exact test.

**Table 2:** Serum sodium levels at admission and prior to surgery.

	Total (*n* = 133)	Control (*n* = 61)	Intestinal Perforation (*n* = 72)	*p*
Sodium at entry (mmol/L)	139 (137 to 141)	139 (136 to 140)	140 (137 to 142)	0.25 ^1^
Sodium bf surgery (mmol/L)	139 (137 to 140)	139 (137 to 141)	139 (136 to 140)	0.37 ^1^
Hosp days bf surgery (days)	1.0 (1.0 to 2.0)	1.0 (1.0 to 3.0)	1.0 (1.0 to 1.0)	<0.001 ^1^

Values are presented as median (IQR). bf = before; Hosp = hospitalization. Test used: ^1^ Mann–Whitney U test.

**Table 3:** Linear mixed model estimates for sodium levels.

	Coefficient (95% CI) (mmol/L)	*p*
Intestinal perforation	0.74 (−0.40 to 1.89)	0.204
Time	0.36 (−0.52 to 1.24)	0.421
Interaction	−1.14 (−2.33 to 0.06)	0.062

## Data Availability

The data presented in this study are available on request from the corresponding author.
